# Establishment of a prognostic ferroptosis‐related gene profile in acute myeloid leukaemia

**DOI:** 10.1111/jcmm.17013

**Published:** 2021-11-05

**Authors:** Ruonan Shao, Huizhong Wang, Wenjian Liu, Jingzi Wang, Shujing Lu, Hailin Tang, Yue Lu

**Affiliations:** ^1^ Sun Yat‐sen University Cancer Center Guangzhou China; ^2^ State Key Laboratory of Oncology in South China Guangzhou China; ^3^ Collaborative Innovation Center for Cancer Medicine Guangzhou China

**Keywords:** acute myeloid leukaemia, ferroptosis, gene profile, prognostic model

## Abstract

Acute myeloid leukaemia (AML) is a heterogeneous disease with a difficult to predict prognosis. Ferroptosis, an iron‐induced programmed cell death, is a promising target for cancer therapy. Nevertheless, not much is known about the relationship between ferroptosis‐related genes and AML prognosis. Herein, we retrieved RNA profile and corresponding clinical data of AML patients from the Gene Expression Omnibus (GEO) and The Cancer Genome Atlas (TCGA) databases. Univariate Cox analysis was employed to identify ferroptosis‐related genes significantly associated with AML prognosis. Next, the least absolute shrinkage and selection operator (LASSO) regression was employed to establish a prognostic ferroptosis‐related gene profile. 12 ferroptosis‐related genes were screened to generate a prognostic model, which stratified patients into a low‐ (LR) or high‐risk (HR) group. Using Kaplan‐Meier analysis, we demonstrated that the LR patients exhibited better prognosis than HR patients. Moreover, receiver operating characteristic (ROC) curve analysis confirmed that the prognostic model showed good predictability. Functional enrichment analysis indicated that the infiltration of regulatory T cells (Treg) differed vastly between the LR and HR groups. Our prognostic model can offer guidance into the accurate prediction of AML prognosis and selection of personalized therapy in clinical practice.

## 1 INTRODUCTION

Acute myeloid leukaemia (AML) has heterogeneous cytogenetic and molecular manifestations. It is distinguished by maturation arrest and unregulated myeloid stem cell proliferation. Advancements in AML treatment, especially in terms of risk classification, combinational chemotherapy and stem cell transplantation, have improved AML outcomes. However, the overall AML prognosis still remains unsatisfactory.^1^, ^2^ The 5‐year survival rate for adult patients with AML is limited to 40%–45% for young patients and is below 10% for aged patients.^3^ Accurate prognostic stratification is essential for the selection of suitable treatment strategies. A number of factors, namely, cytogenetics, molecular genetics and age, are strongly related to AML prognosis, but accurate prognosis prediction is still lacking.^1^, ^4^, ^5^ Hence, additional factors for risk stratification should be identified and combined with analyses of cytogenetic and molecular abnormalities to generate a more precise AML prognostic system.

Iron is indispensable for multiple biological processes, including mitochondrial respiration, immune surveillance, cellular proliferation and metabolic activity.^6^, ^7^ However, massive Iron accumulation in the body can harm cells by inducing membrane lipid peroxidation termed ferroptosis, which is a novel iron‐induced programmed cell death.^8^ Presently, ferroptosis has gained worldwide attention as a potent therapeutic target for cancer therapy. Moreover, several reports have identified genes that modulate AML via ferroptosis.^9^, ^10^ However, the relationship between ferroptosis‐related genes and AML prognosis is currently unknown.

In this report, we retrieved the RNA profile and matching clinical data of AML patients from the Gene Expression Omnibus (GEO) and Cancer Genome Atlas (TCGA) databases. Next, we established a prognostic ferroptosis‐related gene profile in the training cohort (GSE37642‐96). The TCGA‐LAML and GSE37642‐570 cohorts were selected as the external and internal validation cohorts, respectively. Additionally, Gene Set Enrichment Analysis (GSEA) and single‐sample gene set enrichment analysis (ssGSEA) was employed to explore the associated mechanisms. Lastly, a nomogram was generated integrating the risk score (RS) and patient clinical characteristics to estimate AML prognosis.

## 2 MATERIALS AND METHODS

### 2.1 Data collection

The RNA profile and matched patient clinical information of all three AML cohorts were collected from publicly available data sets. GSE37642‐96 and GSE37642‐570 microarray data were retrieved from the GEO database (http://www.ncbi.nlm.nih.gov/geo/) and the TCGA‐LAML RNA‐seq data were obtained from the TCGA website (https://portal.gdc.cancer.gov/repository). The relative gene levels were adjusted with the ‘limma’ R package. In addition, 259 ferroptosis‐related genes were obtained from the FerrDb data set (http://www.zhounan.org/ferrdb/). All data from GEO, TCGA and FerrDb are publicly available, we did not require ethical approval.

### 2.2 Establishment and verification of a prognostic predictive profile

The GSE37642‐96 cohort was selected as the training cohort for the generation of the prognostic model, and, using univariate Cox analysis, ferroptosis‐related genes significantly associated with AML prognosis were identified (P‐value <0.05) in the GSE37642‐96 cohort. Next, the least absolute shrinkage and selection operator (LASSO) regression analysis was used to establish the best ferroptosis‐related gene weighting coefficients. The prognostic model was based on a 10‐fold cross‐validation estimator penalty maximum likelihood estimation. Moreover, the minimum criteria of the penalized maximum likelihood estimator were used to identify the quintessential penalty parameter λ values. The TCGA‐LAML and GSE37642‐570 cohorts were selected as the external and internal validation cohorts, respectively. The RSs of participants were computed with the unified formula established in the training cohort. The patients were then assigned to either a high (HR)‐ or low‐risk (LR) group, based on the median RS of the training cohort.

### 2.3 Quantitative Real‐time polymerase chain reaction

A total of 60 RNA later‐reserved bone marrow specimens were collected from patients who diagnosed AML (30 patients) or non‐haematological malignancies (30 patients) in Sun Yat‐sen University Cancer Center. All samples were stored at −80°C until further analysis. TRIzol reagent (Thermo Fisher Scientific) was used to extract total RNA and PrimeScript™ RT Master Mix (Takara Bio) was used to transcribe RNA into cDNA. Quantitative Real‐time polymerase chain reaction was then performed using a TB Green^®^ Premix Ex Taq (Takara Bio). Student's *t*‐test (two‐tailed) was used for the comparison analyses. The primers used are listed in Table S2.

### 2.4 Functional enrichment analysis

GSEA v4.0.2 software (http://software.broadinstitute.org/gsea/login.jsp) was performed to explore possible biological functions related to the HR and LR cohorts, using the c2.cp.kegg.v7.0.symbols gene sets. NOM *p*‐value <0.05 was considered significant. In addition, we computed the infiltrating score of 16 immune cells and the activity of 13 immune‐linked networks, with ssGSEA.^11^ Gene cloud biotechnology information (GCBI) was employed to assess relationships between model‐linked and other related proteins.

### 2.5 Statistical analysis

Categorical data were analysed by the chi‐square test. Time‐dependent receiver operating characteristic (ROC) curve was done to evaluate the predictive power of ferroptosis‐related gene profile in all cohorts, followed by calculation of the area under the curve (AUC), with the survival ROC package. The confidence interval (CI) was calculated via the bootstrap formula. The inter‐group OS analysis was done via Kaplan‐Meier analysis and log‐rank test. Univariate and multivariate analyses were done to identify independent prognostic factors for AML prognosis. This, in turn, was used to construct the nomogram model to estimate the 1‐, 3‐ and 5‐year OS rates. The calibration plot was adopted to evaluate calibration. R software (Version 3.6.0) or SPSS (Version 24.0) was used for all data analyses. A two‐sided *p* < 0.05 was set as significance threshold.

## 3 RESULTS

### 3.1 Patient characteristics and selection

A total of three cohorts, including 693 AML patients with complete RNA and matched clinical data, were analysed in this study. Patient clinical characteristics are summarized in Table S1. The GSE37642‐96 cohort was selected as the training cohort for the identification of AML prognosis related ferroptosis‐related gene profile, and the TCGA‐LAML and GSE37642‐570 cohorts were selected as the external and the internal validation cohorts, respectively. A summary of our research design is illustrated in Figure S1.

### 3.2 Construction of a prognostic ferroptosis‐related gene signature

**FIGURE 1:**
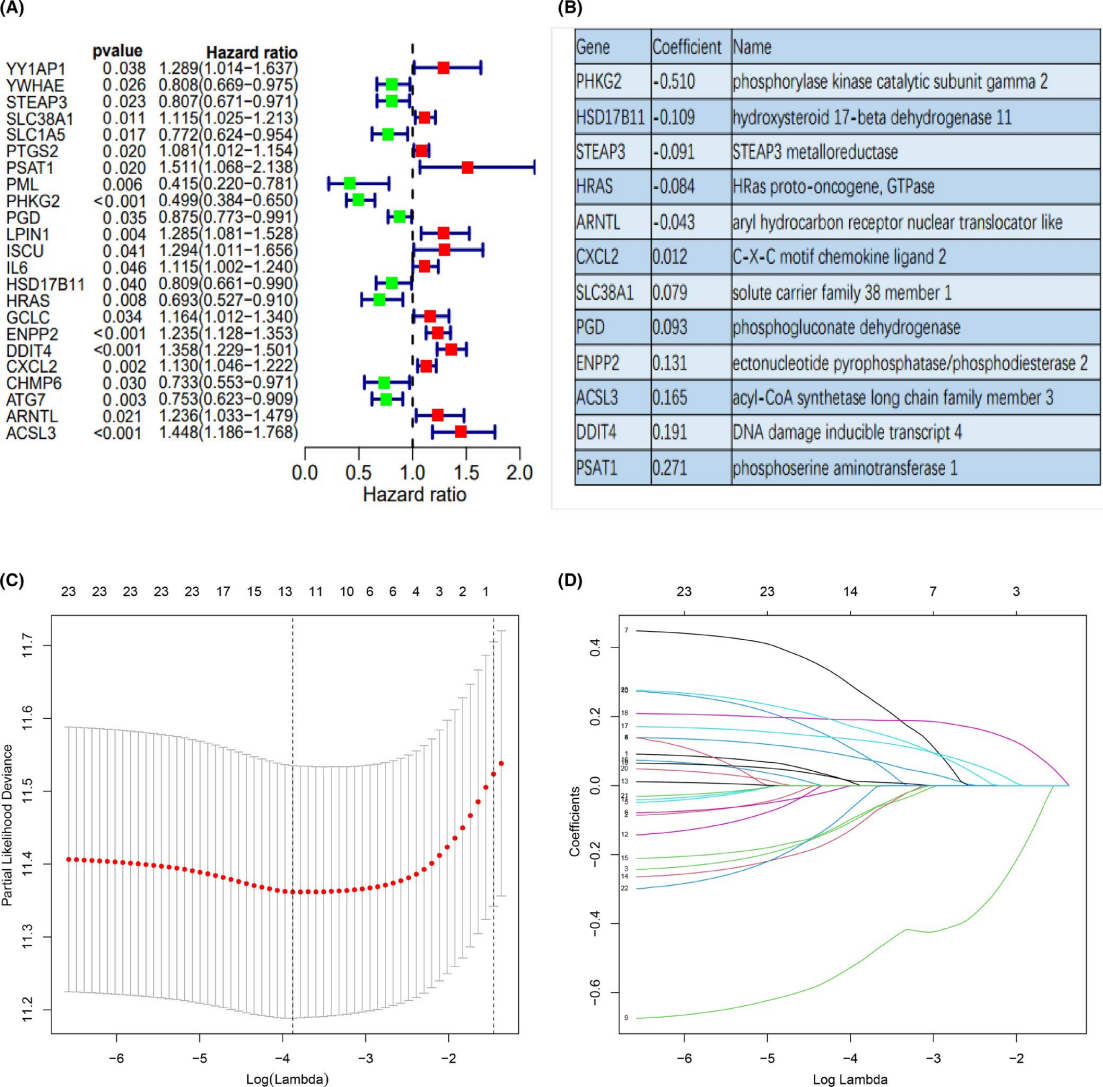
The construction of a prognostic ferroptosis‐related gene profile in the training cohort. (A) Forest plots showing the results of the univariate Cox analysis between gene expression and OS. (B) The corresponding coefficient of 12 ferroptosis‐related genes identified by LASSO regression analysis. (C) 10‐fold cross‐validation for variable selection in the LASSO regression via minimum criteria. (D) LASSO coefficients of ferroptosis‐related genes. Each curve represents a ferroptosis‐related gene

Overall, 210 ferroptosis‐related genes matched with the RNA expression data from three cohorts, were identified. Upon univariate Cox analysis of the 210 ferroptosis‐related genes in the training cohort (GSE37642‐96), 23 genes were considered to correlate with AML overall survival (OS) (*p*‐value <0.05) (Figure 1A). Then, the LASSO regression analysis was applied to construct a ferroptosis‐related gene profile, based on the 23 genes, which are indicative of AML prognosis. Next, based on the penalized maximum likelihood estimator of 1000 bootstrap replicates, a 12‐ferroptosis‐related gene profile was established, with a minimum criteria optimal λ value (Figure 1 C‐D). The formula used for RS computation was as follows: RS = 0.271 × *PSAT1* levels +0.191 × *DDIT4* levels +0.165 × *ACSL3* levels +0.131 × *ENPP2* levels +0.093 × *PGD* levels +0.079 × *SLC38A1* levels +0.012 × *CXCL2* levels – 0.043 × *ARNTL* levels – 0.084 × *HRAS* levels – 0.091 × *STEAP3* levels – 0.109 × *HSD17B11* levels – 0.510 × *PHKG2* levels (Figure 1B).

**FIGURE 2:**
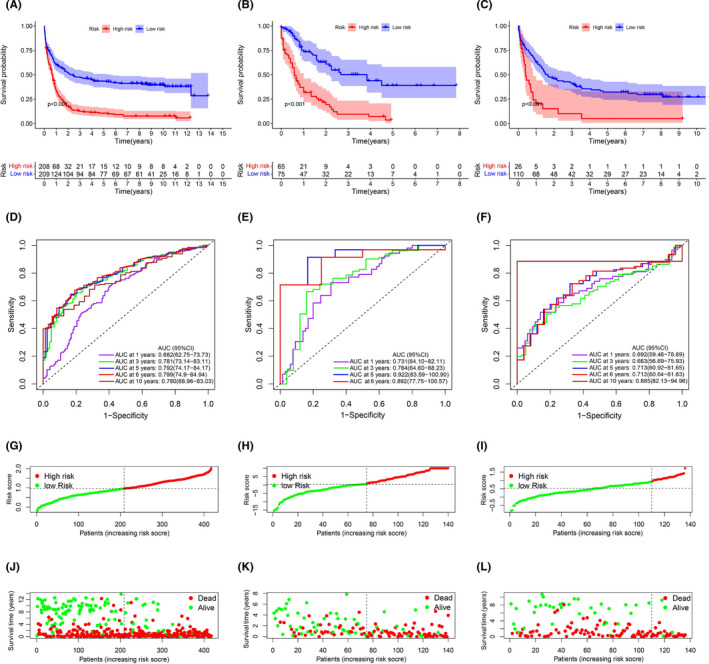
Kaplan–Meier analysis, Time‐dependent ROC analysis, Risk score analysis and Survival outcome analysis for the 12‐ferroptosis‐related gene profile in three cohorts. (A‐C) Kaplan–Meier curve of 12‐ferroptosis‐related gene profile in the training cohort, the TCGA‐LAML cohort and the GSE37642‐570 cohort, respectively. (D‐F) Time‐dependent ROC analysis for 1‐, 3‐, 5‐, 6‐, and 10‐year OS of 12‐ferroptosis‐related gene profile in the training cohort, the TCGA‐LAML cohort and the GSE37642‐570 cohort, respectively. (G‐I) Risk score analysis of the 12‐ferroptosis‐related gene profile in the training cohort, the TCGA‐LAML cohort and the GSE37642‐570 cohort, respectively. (J‐L) Survival outcome analysis of the 12‐ferroptosis‐related gene profile in the training cohort, the TCGA‐LAML cohort and the GSE37642‐570 cohort, respectively

The training cohort was then sub‐categorized into the HR (*n* = 208) or LR sub‐group (*n* = 209), based on the median threshold (Figure 2G). The HR patients were strongly correlated with advanced age, reduced RUNX1_RUNX1T1_fusion, and augmented RUNX1 mutation, relative to the LR patients (Table S1 and Figure 6D). As shown in Figure 2J, the HR patients were also more prone to early demise, compared to the LR patients. In addition, the HR patients exhibited a significantly worse OS, compared to the LR patients (*p *< 0.001) (Figure 2A). We performed Kaplan–Meier analysis in the younger patients (≤60 years) and older patients (>60 years) in the training cohort, those HR patients exhibited a significantly worse OS, compared to the LR patients (*p *< 0.001) in the population ≤60 years and >60 years (Figure S2). In addition, the time‐dependent ROC analysis was employed to assess the sensitivity and specificity of the ferroptosis‐related gene profile. The AUCs for 1‐, 3‐, 5‐, 6‐ and 10‐year OS were 0.682 [95% CI: 62.75−73.73], 0.781 [95% CI: 73.14−83.11], 0.792 [95% CI: 74.17−84.17], 0.799 [95% CI: 74.9−84.94] and 0.760 [95% CI: 68.96−83.03] in the training cohort, respectively (Figure 2D).

### 3.3 Verification of the prognostic ferroptosis‐related gene profile in the two validation cohorts

We assessed the predictability of the ferroptosis‐related gene profile in the external validation cohort (TCGA‐LAML) and the internal validation cohort (GSE37642‐570).

Patients, belonging to the TCGA‐LAML cohort, were stratified into an HR (n = 75) or LR (n = 65) sub‐group, based on the median RS calculated in the training cohort (Figure 2H). The HR patients were strongly correlated with advanced age and higher cytogenetic risk levels, relative to the LR patients (Table S1 and Figure 6E). Consistent with training cohort, the HR patients experienced an enhanced likelihood of death earlier than the LR patients (Figure 2K). In contrast, the LR patients had favourable outcome (*p *< 0.001) (Figure 2B). We performed Kaplan‐Meier analysis in the younger patients (≤60 years) and older patients (>60 years) in the TCGA‐LAML cohort, those LR patients had favourable outcome (*p *< 0.001) in the population ≤60 years and >60 years (Figure S2). In addition, we also performed Kaplan‐Meier analysis in the cytogenetic risk ‘favourable’, ‘median’ and ‘poor’ populations of the TCGA‐LAML cohort, those LR patients had favourable outcome (*p *< 0.001) in the population ‘favourable’, ‘median’ and ‘poor’ (Figure S2). The AUCs for 1‐, 3‐, 5‐ and 6‐year OS were 0.731 [95% CI: 64.10−82.11], 0.764 [95% CI: 64.60−88.23], 0.922 [95% CI: 83.59−100.90] and 0.892 [95% CI: 77.75−100.57] in the TCGA‐LAML cohort, respectively (Figure 2E).

Next, we stratified patients from the GSE37642‐570 cohort into a HR (*n* = 110) or LR (*n* = 26) sub‐group, based on the median RS calculated in the training cohort (Figure 2I). We observed that the HR patients did not have strong associations with clinicopathological characteristics, relative to the LR cohorts (Table S1 and Figure 6F). Moreover, consistent with the training cohort, HR patients experienced death earlier than the LR patients (Figure 2L). Additionally, the LR patients had favourable outcome (*p *< 0.001) (Figure 2C). We performed Kaplan‐Meier analysis in the younger patients (≤60 years) and older patients (>60 years) in the GSE37642‐570 cohort, those LR patients had favourable outcome (*p*< 0.001) in the population ≤60 years and >60 years (Figure S2). The AUCs for 1‐, 3‐, 5‐, 6‐ and 10‐year OS were 0.692 [95% CI: 59.46−78.89], 0.663 [95% CI: 56.69−75.93], 0.713 [95% CI: 60.92−81.65], 0.712 [95% CI: 60.64−81.83] and 0.885 [95% CI: 82.13−94.96] in the GSE37642‐570 cohort, respectively (Figure 2F).

### 3.4 Validation of the expression levels of the 12 ferroptosis‐related genes in clinical specimens

The expression signatures of the 12 ferroptosis‐related genes were explored in 30 AML clinical specimens. The results demonstrated that *HRAS*, *CXCL2*, *SLC38A1*, *PGD*, *ENPP2*, *ACSL3*, *DDIT4* and *PSAT1* mRNA levels were upregulated in AML specimens, while *PHKG2*, *HSD17B11*, *STEAP3* and *ARNTL* was downregulated (Figure S3).

### 3.5 Univariate and multivariate Cox analyses of all three cohorts

**FIGURE 3:**
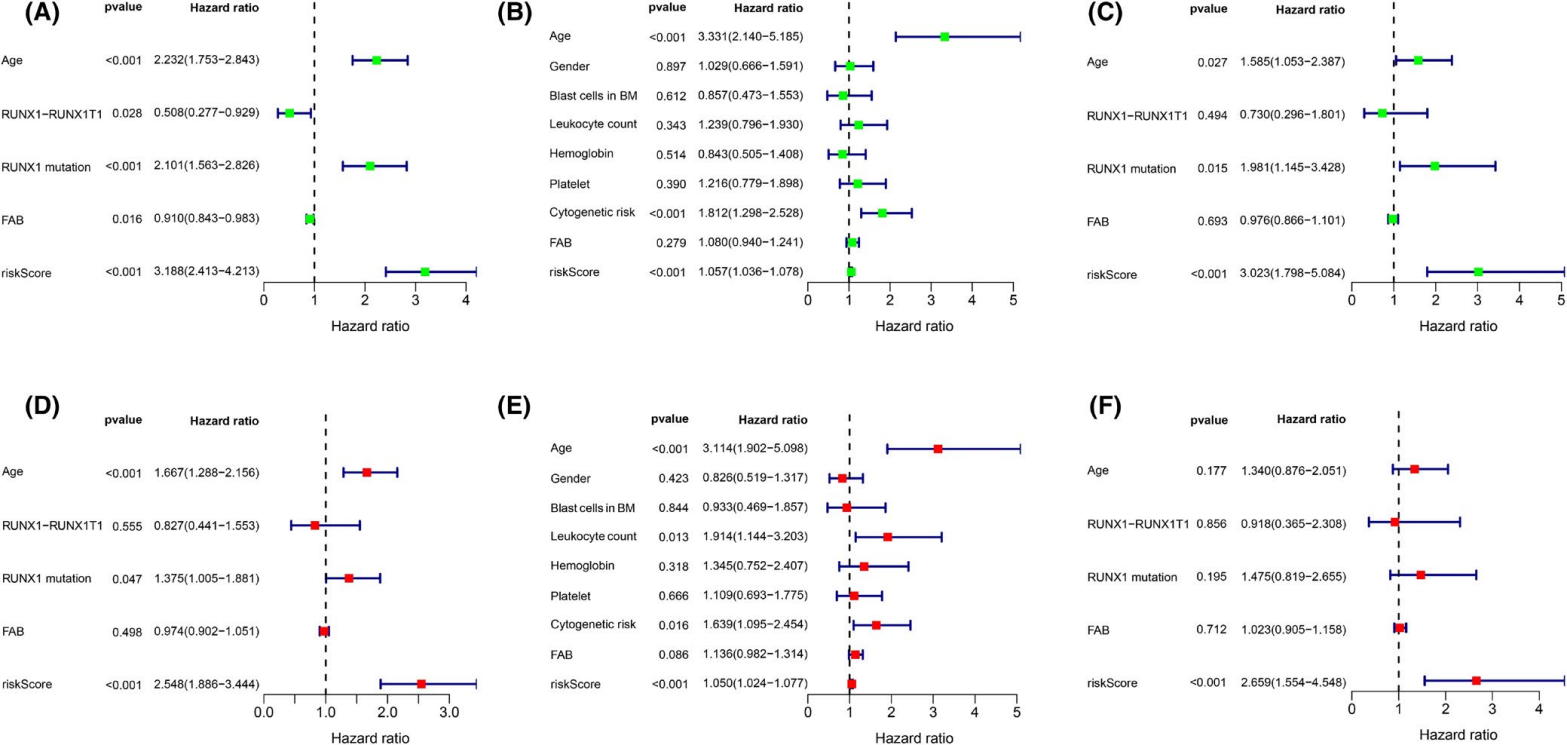
Forrest plots of the univariate and multivariate Cox analysis in three cohorts. Forrest plots of the univariate and multivariate Cox analysis in the training cohort (A, D), the TCGA‐LAML cohort (B, E) and the GSE37642‐570 cohort (C, F)

The univariate and multivariate Cox analyses for RS and other prognostic values were performed in all three cohorts. The univariate Cox analysis indicated that the RS was an independent prognostic indicator for OS in all three cohorts (Figure 3A, B, C). After adjusting other clinical confounding factors in multivariate Cox analysis, the RS was still determined as an independent prognostic predicting factor for AML prognosis in all participants (Figure 3D, E, F).

### 3.6 Functional enrichment analysis in all three cohorts

**FIGURE 4:**
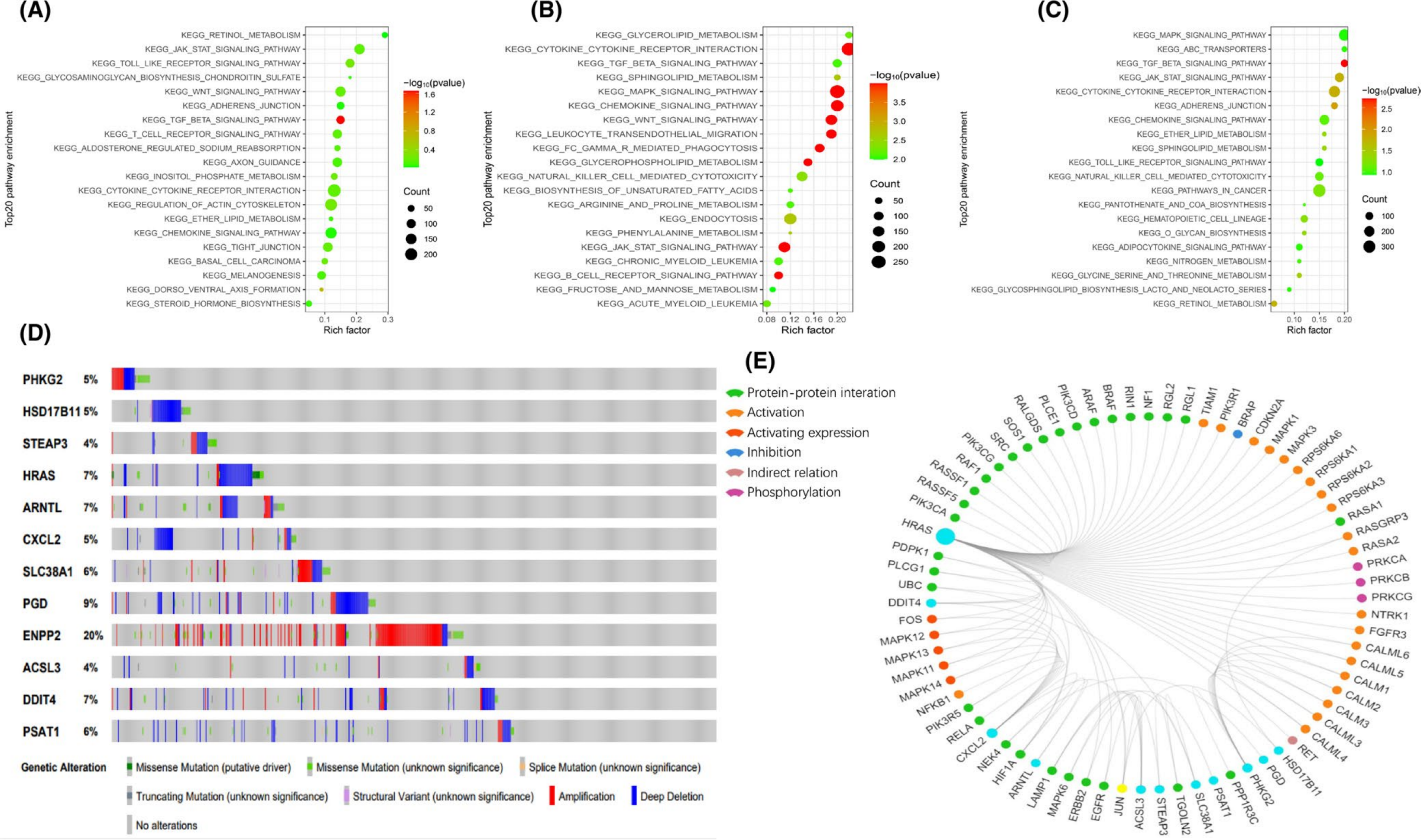
Significantly enriched KEGG pathways in three cohorts by GSEA. Genetic alterations of the 12 ferroptosis‐related genes. (A,B,C) Top 20 representative KEGG pathways in HR group in the training cohort, the TCGA‐LAML cohort and the GSE37642‐570 cohort, respectively (*p *< 0.05). (D) Genetic alterations of the 12 ferroptosis‐related genes in CCLE, obtained from the cBioportal for Cancer Genomics (http://www.cbiop ortal.org/). (E) The protein‐protein interactions between the model related proteins and the other proteins. The model related proteins are shown in blue circles and the size of which is determined by the number of interacting proteins. ENPP2 has no known interactions with other proteins

GSEA was performed in all three cohorts to distinguish the biological functions and networks related to RS. Significantly enriched pathways were detected in the HR population, a majority of which were involved in metabolism (Figure 4A–C). Among these pathways were the fatty acid, glycerophospholipid, ether lipid, phosphoinositol, retinol, phenylalanine metabolism, sphingolipid, arginine, proline, glycine, threonine, fructose, mannose, nitrogen metabolic pathways, unsaturated fatty acids biosynthesis, o‐polysaccharide biosynthesis, steroid hormone biosynthesis and glycosphingolipids biosynthesis pathways. Interestingly, the HR patients also exhibited enrichment in immune‐related biological processes like chemokine, T‐cell receptor, B‐cell receptor, natural killer cell‐mediated cytotoxicity, cytokine‐cytokine receptor interaction, TGF‐β signalling pathways.

**FIGURE 5:**
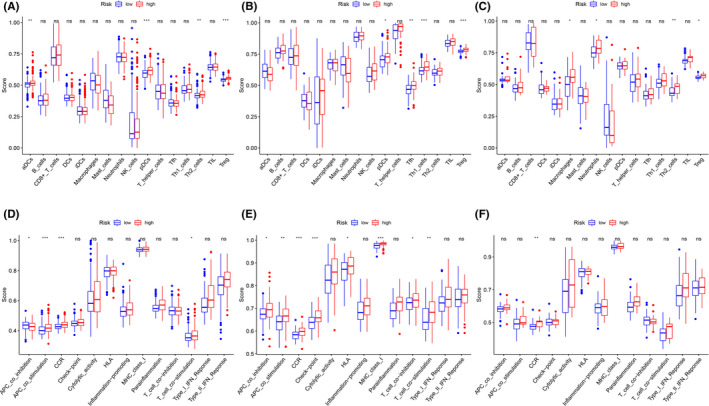
Comparison of the ssGSEA scores between different risk groups in three cohorts. (A‐C) The scores of 16 immune cells in the training cohort, the TCGA‐LAML cohort and the GSE37642‐570 cohort, respectively. (D‐F) 13 immune‐related functions in the training cohort, the TCGA‐LAML cohort and the GSE37642‐570 cohort, respectively. aDC, Activated dendritic cell; APC, Antigen presenting cell; CCR, Cytokine‐cytokine receptor; HLA, Human leukocyte antigen; iDC, Immature dendritic cell; pDC, Plasmacytoid dendritic cell; Tfh, T follicular helper cell; TIL, Tumor Infiltrating Lymphocyte; Treg, Regulatory T cells. Adjusted P values were showed as: ns, not significant; *P< 0.05; ** P< 0.01; *** P< 0.001

To further assess the relationship between RS and immune status, ssGSEA was employed. In total, 16 immune cells with 13 immune‐linked functions were examined. Based on our data, the scores of the aDCs, pDCs, Th2_cells, Treg, APC co‐inhibition, APC co‐stimulation, CCR and T‐cell co‐stimulation were markedly different between the LR and HR patients in the training cohort (*p *< 0.05) (Figure 5A, D). In addition, there were drastic differences in the scores of Tfh, Th1_cells, Treg, APC co‐inhibition, APC co‐stimulation, CCR, check‐point, HLA, MHC class I, T‐cell co‐inhibition and T‐cell co‐stimulation between the LR and HR patients in the TCGA‐LAML population (*p *< 0.05) (Figure 5B, E). Lastly, within the GSE37642‐570 cohort, the scores of macrophages, neutrophils, Th2 cells, Treg and CCR were obviously different between the LR and HR sub‐cohorts (*p *< 0.05) (Figure 5C, F). All three cohorts demonstrated enrichment in the cytokine‐cytokine receptor interaction and, in all three cohorts, the HR patients had an elevated score compared to the LR patients. Lastly, the Treg scores differed between the two risk groups in all three cohorts, suggesting a divergent mechanism.

We next assessed the mutant variants of the ferroptosis‐related gene profile in the Cancer Cell Line Encyclopedia database (CCLE, https://portals.broadinstitute.org/ccle) via the cBioPortal for Cancer Genomics (http://www.cbioportal.org/). Among the 12 ferroptosis‐related genes analysed, gene amplification was the most apparent form of dysregulation (Figure 4D). *ENPP2*, *PGD*, *HRAS*, *ARNTL* and *DDIT4* were commonly altered. We also identified 73 associated proteins from the GCBI database. Among them, eleven were related to our prognostic model, with the exception of *ENPP2* (Figure 4E).

### 3.7 Comparisons of prognostic factors and merged risk scores

**FIGURE 6:**
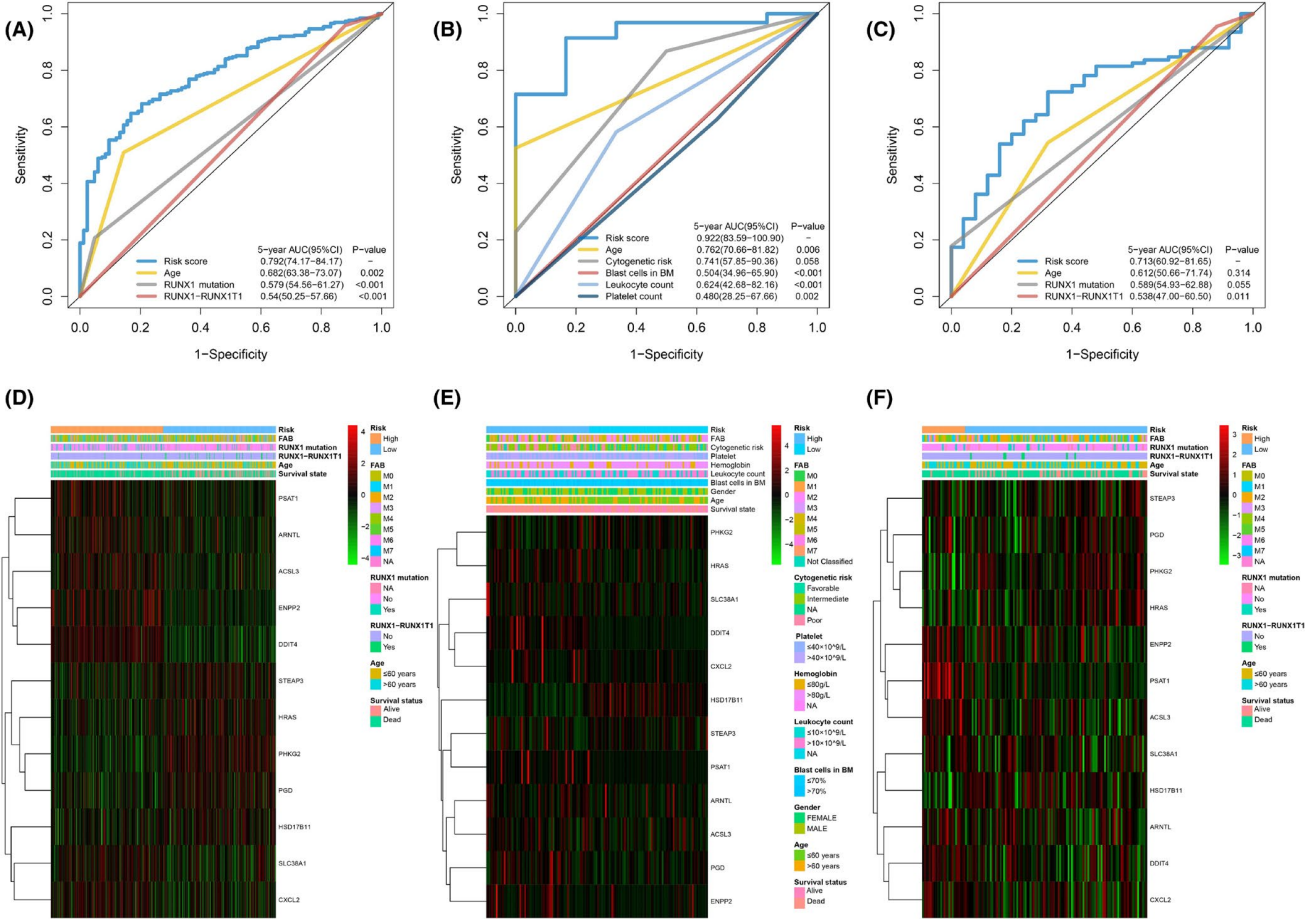
Time‐dependent receiver operating characteristic (ROC) analysis of 5‐year OS of 12‐ferroptosis‐related gene profile compared to other potential prognostic factors in three cohorts. Heatmap of the 12‐ferroptosis‐related gene profile and clinicopathological characteristics in different risk score levels for three cohorts. (A) Time‐dependent ROC analysis for 5‐year OS of 12‐ferroptosis‐related gene profile was compared to age, RUNX1 mutation and RUNX−RUNX1T1in the training cohort. (B) Time‐dependent ROC analysis for 5‐year OS of 12‐ferroptosis‐related gene profile was compared to age, cytogenetic risk, blast cells in bone marrow, platelet, and leukocyte count in the TCGA‐LAML cohort. (C) Time‐dependent ROC analysis for 5‐year OS of 12‐ferroptosis‐related gene profile was compared to age, RUNX1‐RUNX1T1, and RUNX1 mutation in the GSE37642‐570 cohort. (D‐F) Heatmap of the 12‐ferroptosis‐related gene profile and clinicopathological characteristics in different risk score levels for the training cohort (D), the TCGA‐LAML cohort (E) and the GSE37642‐570 cohort (F), respectively

Compared to other potential prognostic values, the prognostic sensitivity and specificity of the ferroptosis‐related gene profile were powerful. The AUC (0.78 [95% CI: 0.73–0.83]) of the RS for the 5‐year OS was markedly elevated, compared to other values like age (0.66 [95% CI: 0.61–0.71]), RUNX1‐RUX1T1 fusion (0.47 [95% CI: 0.43 – 0.49]) and RUNX1 mutation (0.58 [95% CI: 0.56–0.61]) in the training cohort (all *p* < 0·001, Figure 6A). Consistent with the training cohort, the AUC of the RS for the 5‐year OS was also markedly elevated, compared to other values in the TCGA‐LAML population (Figure 6B). Additionally, the RS revealed a numerically but not statistically larger AUC value for the 5‐year OS, relative to the age in the GSE37642‐570 cohort (Figure 6C).

**FIGURE 7:**
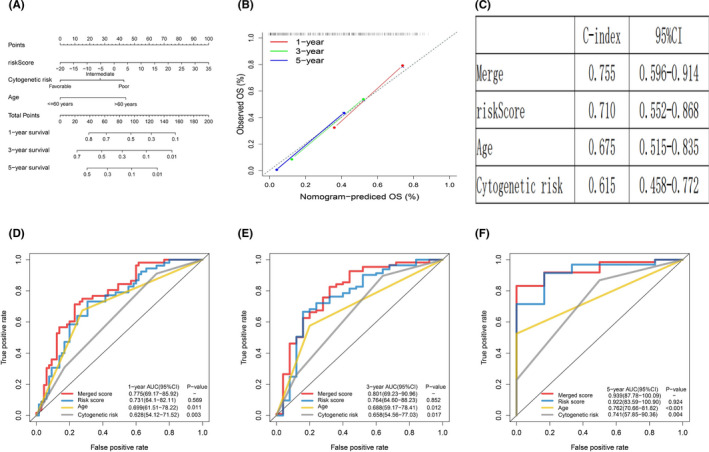
Building and validation of the nomogram to predict the OS of AML patients in the TCGA‐LAML cohort. (A) Nomogram was built based on age, cytogenetic risk and risk score in the TCGA‐LAML cohort. (B) Calibration plot of the nomogram. (C) C‐index of the nomogram. (D‐F) Time‐dependent receiver operating characteristic (ROC) curves of nomograms were compared based on 1‐, 3‐, and 5‐year OS of the TCGA‐LAML cohort

Nomogram, a highly precise assessment technology, was employed for the integration of age, cytogenetic risk category and ferroptosis‐related gene profile in the TCGA‐LAML cohort (Figure 7A). Moreover, the calibration plots revealed that the nomogram can estimate the 1‐, 3‐ and 5‐year OS with great precision (Figure 7B). The C‐index for the nomogram was 0.755 (Figure 7C). The 1‐, 3‐ and 5‐year OS AUC of merged score was considerably higher than the age and cytogenetic risk category, suggesting that the nomogram can enhance the OS prediction, relative to the standard prognostic factors (Figure 7D‐F).

## 4 DISCUSSION

Currently, AML remains among the most complicated and challenging diseases.^12^ Despite the development of novel drugs and risk‐stratified therapies, the 5‐year OS rate is still below 40%, owing to the heterogeneous nature of this disease.^13^ Hence, it is urgently required to identify new prognostic indicators to enhance the diagnosis, personalized therapy and prognosis of AML patients. Ferroptosis, a newly discovered ferroptosis‐induced programmed cell death, with associated iron accumulation and lipid peroxidation, is essential for eradicating carcinogenic cells.^14^, ^15^ Targeting ferroptosis has emerged as a promising anticancer therapy. In fact, some previous studies have suggested that leukaemia is susceptible to ferroptosis and erastin, an inducer of ferroptosis, can enhance the anticancer activity of cytarabine and doxorubicin by enhancing ferroptosis.^16^, ^17^ Moreover, several genes are known to modulate ferroptosis in AML.^9^, ^10^ Nevertheless, there are no current reports on the relationship between ferroptosis‐related genes and AML prognosis.

Herein, we employed univariate Cox and LASSO regression analyses to identify OS‐related ferroptosis‐related genes in AML samples. A new model for predicting AML prognosis was then generated using 12 ferroptosis‐related genes in the training cohort (GSE37642‐96), and was further verified in two validation cohorts, namely, the external validation cohort (TCGA‐LAML) and the internal validation cohort (GSE37642‐570). Next, using the prognostic model, patients were stratified into HR (poor prognosis) or LR (favourable prognosis) sub‐group. In addition, a stably enhanced prognostic value was achieved for AML. Subsequently, we generated a nomogram integrating RS and historical prognostic factors like age and cytogenetic risk, to form precise OS predictions for AML patients. Lastly, using functional analysis, we demonstrated that metabolism and immune‐associated pathways were highly enriched in the HR versus LR sub‐cohort.

Among the 12 ferroptosis‐related genes (*PHKG2*, *HSD17B11*, *STEAP3*, *HRAS*, *ARNTL*, *CXCL2*, *SLC38A1*, *PGD*, *ENPP2*, *ACSL3*, *DDIT4* and *PSAT1*), which were used to construct the prognostic model in this study, most have been reported to regulate ferroptosis progression via modulation of lipid oxidation and energy metabolism. *PHKG2* is a regulator of polyunsaturated fatty acid peroxidation during ferroptosis, and silencing *PHKG2* attenuates U‐2‐OS cell sensitivity to Erastin.^18^ *HSD17B11* is an enzyme involved in reduction or oxidation of sex hormones and is reported to be enriched in RSL3‐resistant cells.^19^ *STEAP3*, a metalloreductase capable of converting iron from Fe3+ to Fe2+, participates in the processes of *TP53*‐mediated regulation of the transcription of cell death genes and ferroptosis.^20^ *HRAS*, originating from cell surface receptors, is a crucial member of multiple cellular signalling networks. *RAS* mutations occur in 25% of AML patients and enhance the sensitivity to cytarabine.^21^ It is reported to be involved in modulating cancer cells sensitivity to oxidative stress. However, the expression of oncogenic *RAS* in different cancers presented distinct responses (sensitive or resistant) to ferroptosis inducers.^22^, ^23^, ^24^ Therefore, the relation between oncogenic *RAS* and ferroptosis is still controversial. *ARNTL* suppresses ferroptosis via repression of *EGLN2* transcription and activation of the prosurvival transcription factor *HIF1A*.^25^ Some previous findings suggested that the overexpression of *ARNTL* showed improved sensitivity to anticancer drugs.^26^, ^27^ *SLC38A1* is an essential mediator of glutamine uptake and metabolism in lipid peroxidation and *SLC38A1* knockout can markedly block ferroptosis.^28^ However, previous study confirmed that high expression of *SLC38A1* was an adverse prognostic factor for AML.^29^ *ENPP2* is a lipid kinase involved in the lipid metabolic pathway, and it protects cardiomyocytes from erastin‐driven ferroptosis.^30^ *ACSL3* catalyses the conversion of exogenous monounsaturated fatty acids into fatty acyl‐CoAs and is necessary for fatty acids activation and cell resistance to ferroptosis.^31^ *PGD*, a cytosolic enzyme belonging to the carbohydrate metabolism pathway, is essential for ER structural integrity and protein secretion.^32^ In summary, three of the above‐mentioned genes (*PHKG2*, *STEAP3* and *SLC38A1*) promote ferroptosis, whereas three genes (*ARNTL*, *ENPP2* and *ACSL3*) prevent ferroptosis. However, whether these genes modulate ferroptosis and impact AML prognosis needs further investigation.

Despite extensive research on the mechanisms underlying tumour susceptibility to ferroptosis, not much is known about the relationship between tumour immunity and ferroptosis. To fill this gap, we conducted GSEA analysis and surprisingly found that numerous immune‐linked biological processes and pathways were enriched, which indicates a possible connection between ferroptosis and tumour immunity. Nevertheless, such a connection has rarely been reported. Interestingly, the ssGSEA analysis revealed that the HR patients in all three cohorts exhibit elevated Treg. Prior reports have suggested that Treg cells modulate immune escape via suppression of antileukaemia activity. In addition, a high fraction of Treg in the tumour microenvironment is associated with progression and poor survival for haematological malignancies.^33^, ^34^ We, therefore, speculated that the poor prognosis of the HR group might be related to more Treg infiltration in the immune microenvironment. Moreover, some previous studies suggested that the induction of ferroptosis tumour cells at early stage may efficiently enhance immune response and the inducing of Treg cell ferroptosis may be a therapeutic strategy to improve cancer treatment.^35^, ^36^ So the use of ferroptosis inducers may help improve the patients' therapeutic effect in the HR group.

Although our prognostic model exhibited better predictive power than traditional prognostic factors, certain study limitations must be considered. Our prognostic model was based on publicly available retrospective data. Hence, additional prospective investigations are needed to validate its predictive power. Second, the underlying mechanisms between ferroptosis and AML progression remain poorly understood and further examinations are required to elucidate the importance of ferroptosis‐related genes in AML pathology.

## 5 CONCLUSION

We constructed a novel prognostic model with 12 ferroptosis‐related genes and a nomogram combined the RS, age and cytogenetic risk category in AML. We also demonstrated that our prognostic model and nomogram have high accuracy in predicting OS, which indicates that the ferroptosis‐related gene profile can possibly be employed as an indicator of AML diagnosis and prognosis. Furthermore, the prognostic performance of our prognostic model and nomogram can offer guidance towards the accurate prediction and personalized therapy of AML patients in clinical practice.

## CONFLICT OF INTEREST

The authors declare no conflicts of interest in this work.

## AUTHOR CONTRIBUTIONS

**Ruonan Shao:** Data curation (equal); Formal analysis (equal); Investigation (equal); Methodology (equal); Software (equal); Validation (equal); Visualization (equal); Writing‐original draft (equal). **Huizhong Wang:** Data curation (equal); Formal analysis (equal); Investigation (equal); Methodology (equal); Software (equal); Validation (equal); Visualization (equal); Writing‐original draft (equal). **Wenjian Liu:** Data curation (equal); Formal analysis (equal); Investigation (equal); Methodology (equal); Software (equal); Validation (equal); Visualization (equal); Writing‐original draft (equal). **Jingzi Wang:** Investigation (equal); Methodology (equal); Software (equal); Visualization (equal). **Shujing Lu:** Investigation (equal); Methodology (equal); Software (equal); Validation (equal). **Hailin Tang:** Funding acquisition (equal); Project administration (equal); Supervision (equal); Writing‐original draft (equal); Writing‐review & editing (equal). **Yue Lu:** Funding acquisition (equal); Project administration (equal); Supervision (equal); Writing‐original draft (equal); Writing‐review & editing (equal).

## Supporting information

Fig S1

Fig S2

Fig S3

Table S1

Table S2

## Data Availability

All data from GEO, TCGA and FerrDb are publicly available.
