# Supplementary figures and images for: Establishment of a prognostic ferroptosis‐related gene profile in acute myeloid leukaemia
Source: J Cell Mol Med. 2021 Nov 5;25(23):10950–60. doi: 10.1111/jcmm.17013 (PMC8642683; doi:10.1111/jcmm.17013)

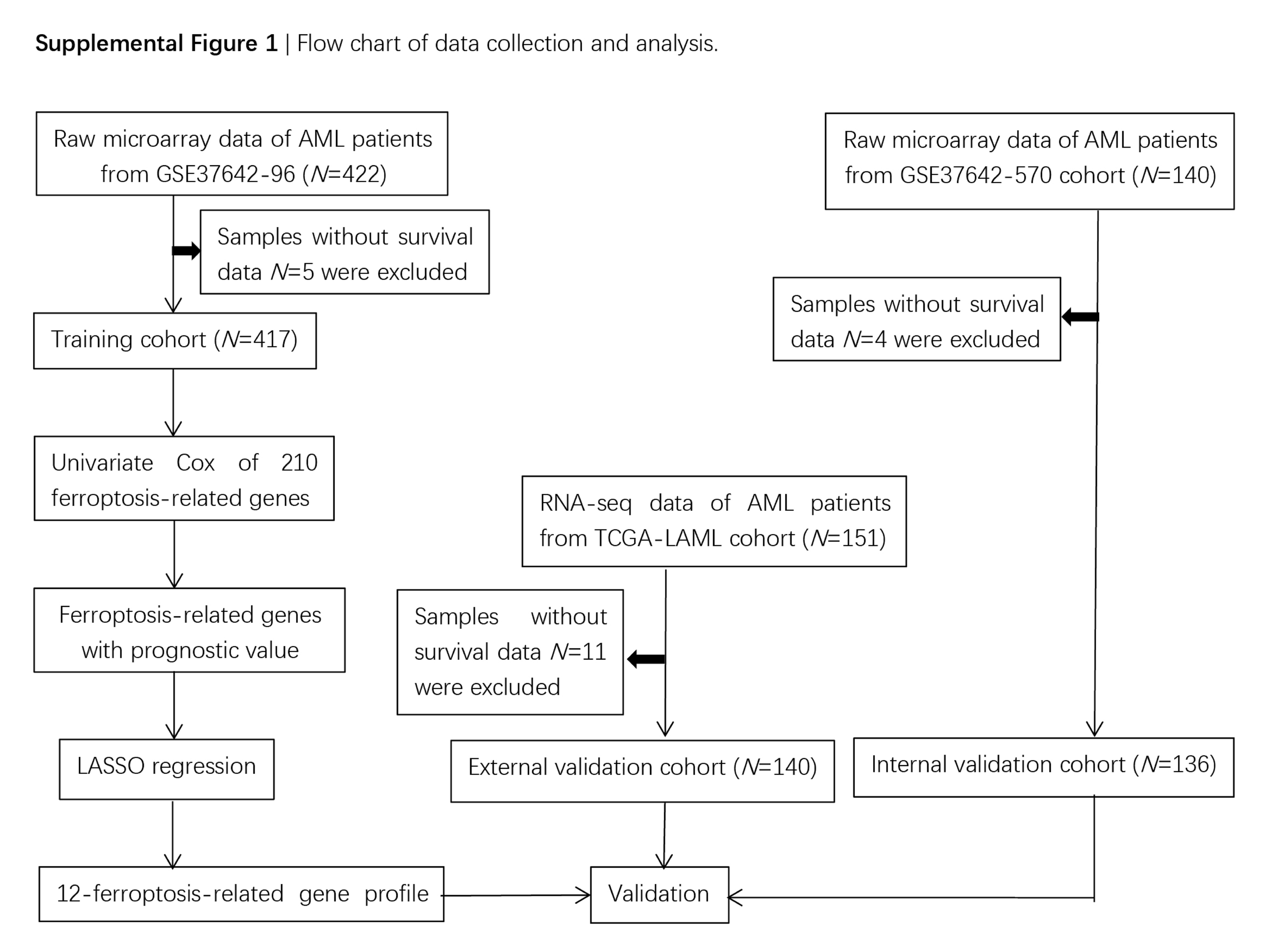

Supplement: Supplementary file 1 — Fig S1 [file JCMM-25-10950-s003.jpg]

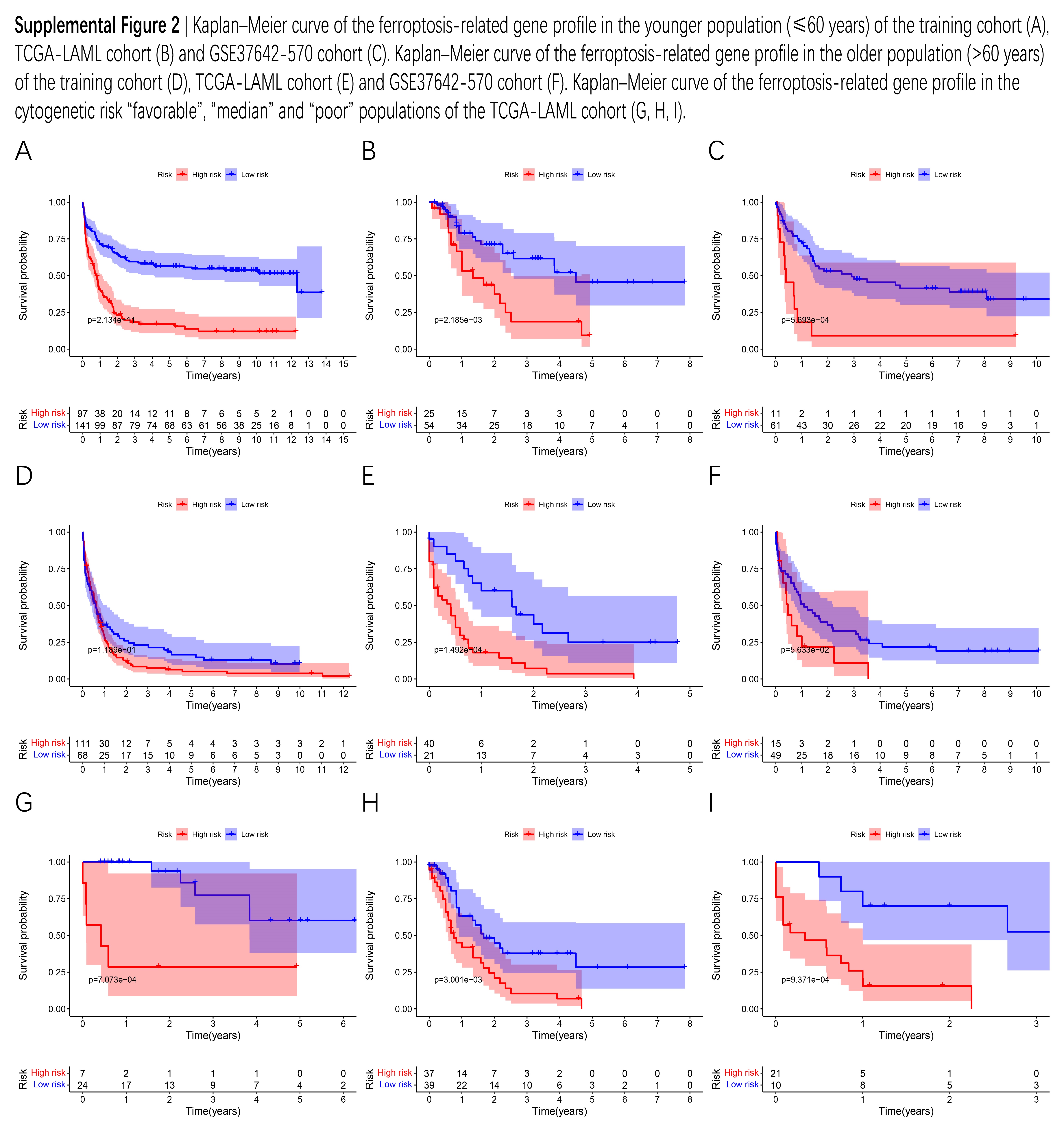

Supplement: Supplementary file 2 — Fig S2 [file JCMM-25-10950-s001.jpg]

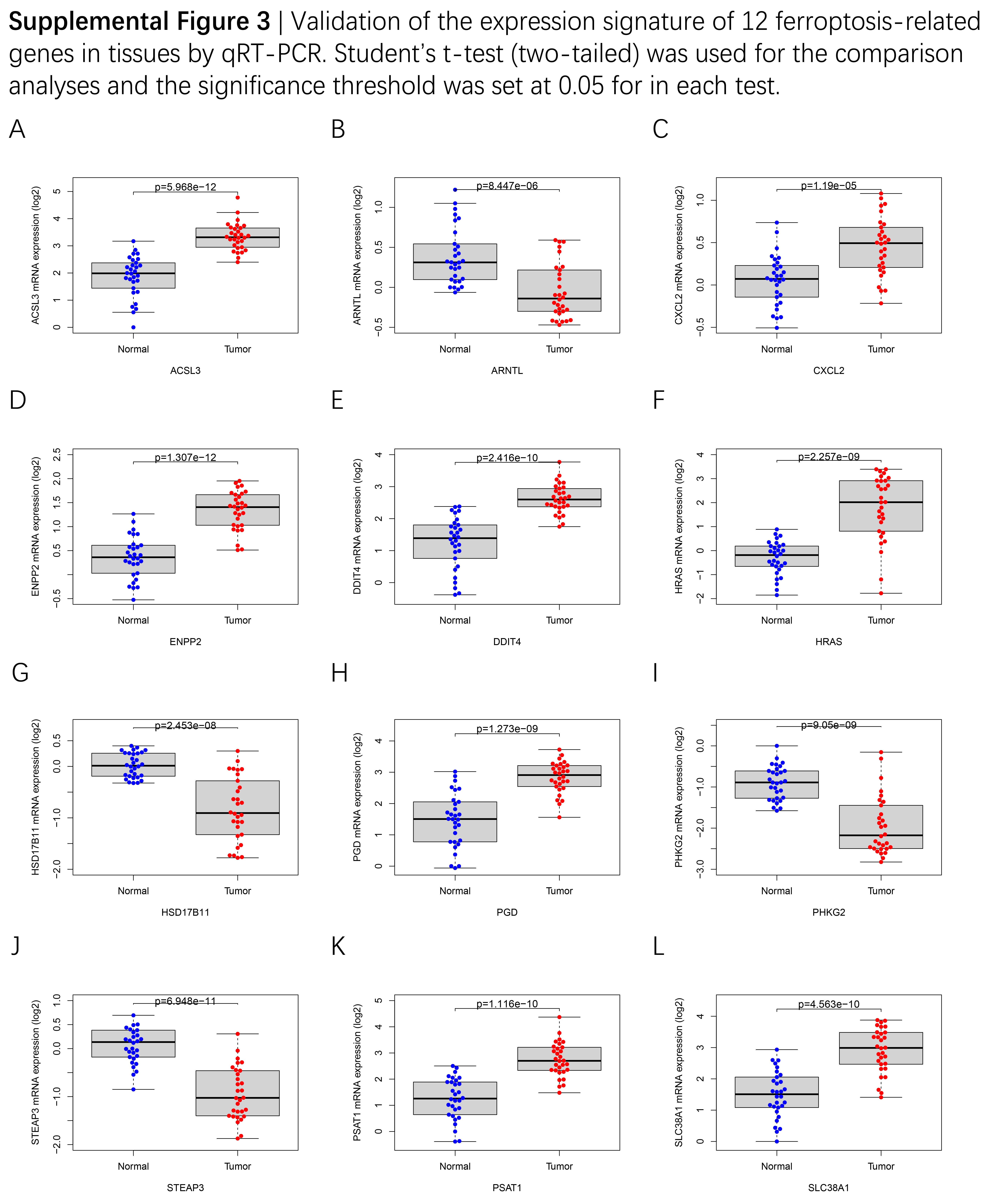

Supplement: Supplementary file 3 — Fig S3 [file JCMM-25-10950-s002.jpg]
