# Supplementary material for: Establishment of a prognostic ferroptosis‐related gene profile in acute myeloid leukaemia
Source: J Cell Mol Med. 2021 Nov 5;25(23):10950–60. doi: 10.1111/jcmm.17013 (PMC8642683; doi:10.1111/jcmm.17013)
Supplement: Supplementary file 4 — Table S1 [file JCMM-25-10950-s005.docx]

**Supplemental Table 1** | Clinical and molecular characteristics of AML patients according to risk score levels in the training cohort, the external validation cohort, and the internal validation cohort, respectively.

|  |  | **Low risk (%)** | **High risk (%)** | **P-value** |
| --- | --- | --- | --- | --- |
| **Training cohort  (GSE37642-96, *N* = 417)** |  | **209** | **208** |  |
| Age |  |  |  |  |
|  | ≤60 years | 141 (67.5) | 97 (46.6) | < 0.001 |
|  | > 60 years | 68 (32.5) | 111 (53.4) |  |
| RUNX1-RUNX1T1 | |  |  |  |
|  | No | 189 (90.4) | 205 (98.6) | < 0.001 |
|  | Yes | 20 (9.6) | 3 (1.4) |  |
| RUXN1 mutationa | |  |  |  |
|  | Neg | 162 (91.0) | 149 (77.6) | < 0.001 |
|  | Pos | 16 (9.0) | 43 (22.4) |  |
| FAB subtypeb | |  |  |  |
|  | M0 | 2 (1.0) | 12 (5.9) | < 0.001 |
|  | M1 | 28 (14.0) | 56 (27.7) |  |
|  | M2 | 67 (33.5) | 50 (24.8) |  |
|  | M3 | 18 (9.0) | 1 (0.5) |  |
|  | M4 | 54 (27.0) | 50 (24.8) |  |
|  | M5 | 26 (13.0) | 21 (10.4) |  |
|  | M6 | 5 (2.5) | 10 (5.0) |  |
|  | M7 | 0 (0.0) | 2 (1.0) |  |
| **External validation cohort  (TCGA, *N* = 140)** |  | **75** | **65** |  |
| Age |  |  |  |  |
|  | ≤60 years | 54 (72.0) | 25 (38.5) | <0.001 |
|  | > 60 years | 21 (28.0) | 40 (61.5) |  |
| Gender | |  |  |  |
|  | Male | 38 (49.3) | 39 (60.0 | 0.268 |
|  | Female | 37 (50.7) | 26 (40.0) |  |
| Blast cells in BM (%) | |  |  |  |
|  | ≤ 70 | 58(77.3) | 55 (84.6) | 0.276 |
|  | > 70 | 17 (22.7) | 10 (15.4) |  |
| Leukocyte count (x10^9/L)c | | |  |  |
|  | ≤ 10 | 27 (36.5) | 29 (44.6) | 0.330 |
|  | > 10 | 47 (63.5) | 36 (55.4) |  |
| Hemoglobin (mg/dL)^d^ | |  |  |  |
|  | ≤ 80 | 14 (18.9) | 13(20.0) | 0.872 |
|  | > 80 | 60 (81.1) | 52 (80.0) |  |
| Platelet count (x10^9/L) | |  |  |  |
|  | ≤ 40 | 36 (48.0) | 26 (40.0) | 0.342 |
|  | > 40 | 39 (52.0) | 39 (60.0) |  |
| Cytogenetic risk^e^ | |  |  |  |
|  | Faverable | 24(32.9) | 7 (10.8) | 0.002 |
|  | Intermediate | 39 (53.4) | 37 (56.9) |  |
|  | Poor | 10 (13.7) | 21 (32.3) |  |
| FAB subtype | |  |  |  |
|  | M0 | 5 (6.7) | 9(13.8) | 0.059 |
|  | M1 | 14 (18.7) | 16(24.6) |  |
|  | M2 | 21 (28) | 13 (20.0) |  |
|  | M3 | 10 (13.3) | 5 (7.7) |  |
|  | M4 | 20 (26.7) | 8 (12.3) |  |
|  | M5 | 5 (6.7) | 10 (15.4) |  |
|  | M6 | 0 (0.0) | 2 (3.1) |  |
|  | M7 | 0 (0.0) | 1 (1.5) |  |
|  | Not Classified | 0 (0.0) | 1 (1.5) |  |
| **Internal validation cohort  (GSE37642-570, *N* = 136)** |  | **110** | **26** |  |
| Age |  |  |  |  |
|  | ≤60 years | 61 (55.5) | 11 (42.3) | 0.227 |
|  | > 60 years | 49 (44.5) | 15 (57.7) |  |
| RUNX1-RUNX1T1 | |  |  |  |
|  | No | 103(93.6) | 26 (100) | 0.187 |
|  | Yes | 7 (6.4) | 0 (0.0) |  |
| RUXN1 mutation^f^ | |  |  |  |
|  | Neg | 86 (86.9) | 22 (88.0) | 0.602 |
|  | Pos | 13(13.1) | 3 (12.0) |  |
| FAB subtype^g^ | |  |  |  |
|  | M0 | 7 (6.4) | 1 (3.8) | 0.688 |
|  | M1 | 24 (22.0) | 5 (19.2) |  |
|  | M2 | 38 (34.9) | 9 (34.6) |  |
|  | M3 | 7 (6.4) | 0 (0) |  |
|  | M4 | 14 (12.8) | 3 (11.5) |  |
|  | M5 | 14 (12.8) | 5 (19.2) |  |
|  | M6 | 4 (3.7) | 3 (11.5) |  |
|  | M7 | 1 (0.9) | 0 (0.0) |  |

a-g. missing data of 47, 15, 1, 1, 2, 12 and 1 patients, respectively.
