# Supplementary material for: Establishment of a prognostic ferroptosis‐related gene profile in acute myeloid leukaemia
Source: J Cell Mol Med. 2021 Nov 5;25(23):10950–60. doi: 10.1111/jcmm.17013 (PMC8642683; doi:10.1111/jcmm.17013)
Supplement: Supplementary file 5 — Table S2 [file JCMM-25-10950-s004.docx]

| *PHKG2* | Forward Primer | GAGTTTTACCAGAAGTACGACCC |
| --- | --- | --- |
|  | Reverse Primer | GTAGCTCGATGAACACAACGG |
| *HSD17B11* | Forward Primer | CCTGCTTCTCCCGTTACTGAT |
|  | Reverse Primer | GATTTCGCCGGTGACTGATTT |
| *STEAP3* | Forward Primer | CGGAGGTCATCTTTGTGGCTGTG |
|  | Reverse Primer | GCTGAAGGTGCTCTTGCTCTGTAG |
| *HRAS* | Forward Primer | ATGACGGAATATAAGCTGGTGGT |
|  | Reverse Primer | GGCACGTCTCCCCATCAATG |
| *ARNTL* | Forward Primer | AAGGGAAGCTCACAGTCAGAT |
|  | Reverse Primer | GGACATTGCGTTGCATGTTGG |
| *CXCL2* | Forward Primer | CGAAGTCATAGCCACACTCAAG |
|  | Reverse Primer | CTTCTGGTCAGTTGGATTTGC |
| *SLC38A1* | Forward Primer | GCTTTGGTTAAAGAGCGGGC |
|  | Reverse Primer | CTGAGGGTCACGAATCGGAG |
| *PGD* | Forward Primer | ATGGCCCAAGCTGACATCG |
|  | Reverse Primer | AAAGCCGTGGTCATTCATGTT |
| *ENPP2* | Forward Primer | ACTTTTGCCGTTGGAGTCAAT |
|  | Reverse Primer | GGAGTCTGATAGCACTGTAGGA |
| *ACSL3* | Forward Primer | AGGAGGTCCAGCCATTGTTC |
|  | Reverse Primer | CTATGAGGTTGGTTTTCCATGCT |
| *DDIT4* | Forward Primer | TGAGGATGAACACTTGTGTGC |
|  | Reverse Primer | CCAACTGGCTAGGCATCAGC |
| *PSAT1* | Forward Primer | TGCCGCACTCAGTGTTGTTAG |
|  | Reverse Primer | GCAATTCCCGCACAAGATTCT |

**Supplementary Table 2** | qRT-PCR Primers for 12 ferroptosis-related genes
